# A kinetic fluorescence assay reveals unusual features of Ca^++^ uptake in *Plasmodium falciparum*-infected erythrocytes

**DOI:** 10.1186/1475-2875-13-184

**Published:** 2014-05-18

**Authors:** Elizabeth M Zipprer, McKinzie Neggers, Ambuj Kushwaha, Kempaiah Rayavara, Sanjay A Desai

**Affiliations:** 1 The Laboratory of Malaria and Vector Research, National Institute of Allergy and Infectious Diseases, National Institutes of Health, Bethesda, MD 20892, USA

**Keywords:** Calcium channels, Malaria parasites, Human erythrocytes, Fluo-8, Fluorescence kinetics, Antimalarial drug discovery

## Abstract

**Background:**

To facilitate development within erythrocytes, malaria parasites increase their host cell uptake of diverse solutes including Ca^++^. The mechanism and molecular basis of increased Ca^++^ permeability remains less well studied than that of other solutes.

**Methods:**

Based on an appropriate Ca^++^ affinity and its greater brightness than related fluorophores, Fluo-8 was selected and used to develop a robust fluorescence-based assay for Ca^++^ uptake by human erythrocytes infected with *Plasmodium falciparum*.

**Results:**

Both uninfected and infected cells exhibited a large Ca^++^-dependent fluorescence signal after loading with the Fluo-8 dye. Probenecid, an inhibitor of erythrocyte organic anion transporters, abolished the fluorescence signal in uninfected cells; in infected cells, this agent increased fluorescence via mechanisms that depend on parasite genotype. Kinetic fluorescence measurements in 384-well microplates revealed that the infected cell Ca^++^ uptake is not mediated by the plasmodial surface anion channel (PSAC), a parasite nutrient channel at the host membrane; it also appears to be distinct from mammalian Ca^++^ channels. Imaging studies confirmed a low intracellular Ca^++^ in uninfected cells and higher levels in both the host and parasite compartments of infected cells. Parasite growth inhibition studies revealed a conserved requirement for extracellular Ca^++^.

**Conclusions:**

Nondestructive loading of Fluo-8 into human erythrocytes permits measurement of Ca^++^ uptake kinetics. The greater Ca^++^ permeability of cells infected with malaria parasites is apparent when probenecid is used to inhibit Fluo-8 efflux at the host membrane. This permeability is mediated by a distinct pathway and may be essential for intracellular parasite development. The miniaturized assay presented here should help clarify the precise transport mechanism and may identify inhibitors suitable for antimalarial drug development.

## Background

While intracellular development of malaria parasites in vertebrate erythrocytes protects the parasite from host immune response and permits digestion of haemoglobin as an amino acid source [1], it also limits access to nutrients and ions in serum. To overcome this limitation, malaria parasites increase erythrocyte permeability to diverse solutes, including essential nutrients, halide anions, organic cations, and inorganic cations such as K^+^ and Ca^++^[2-10]. Remarkably, Na^+^ permeability is increased to a much lesser extent, paralleling a requirement for exclusion of this ion to maintain osmotic stability of the erythrocyte [11-13].

The increased uptake of most solutes is mediated by a parasite-derived ion channel, known as the plasmodial surface anion channel (PSAC) [14-17], but additional channels or modified host transporters may contribute for specific solutes [18,19]. Increased Ca^++^ permeability is important because this divalent cation has a remarkably low concentration in erythrocyte cytosol [20], but is nevertheless required for intracellular parasite development [21-23]. Increased Ca^++^ uptake does not reflect inhibition of the erythrocyte’s Ca^++^ ATPase pump or upregulation of an endogenous divalent cation carrier [24]; lack of inhibition by furosemide, a nonspecific PSAC inhibitor [25], suggests Ca^++^ uptake is not mediated by PSAC [8].

The precise mechanism of Ca^++^ uptake after infection is unclear, with proposals including activation of an erythrocyte-specific transport mechanism, leakiness due to experimental manipulation, or a channel of parasite origin [8,24,26]. The absence of simple methods for measuring changes in Ca^++^ uptake after infection has limited progress. A fluorescence based-assay has, therefore, been developed to detect and track the increased Ca^++^ permeability of infected cells in a miniaturized 384-well microplate format. The assay implicates low rates of transport at the host membrane and suggests an improved model of Ca^++^ trafficking within infected erythrocytes. It has also been used to determine that mammalian Ca^++^ channel blockers and a highly specific PSAC inhibitor are inactive against infected cell Ca^++^ uptake. This new cell-based assay should assist in identifying inhibitors and characterizing the responsible transport mechanisms.

## Methods

### Parasite culture

The Dd2, HB3, and 3D7A *Plasmodium falciparum* lines were cultivated separately in RPMI 1640 medium (Life technologies, Grand Island, NY, USA) with 0.5% NZ microbiological BSA (MP Biomedicals, Santa Ana, CA, USA) using O^+^ human erythrocytes obtained from anonymous donors (Interstate Blood Bank, Memphis, TN, USA). Cultures were harvested at the trophozoite stage and enriched to > 95% parasitaemia by the percoll-sorbitol method [27,28].

### Dye loading and fluorescence measurements

Uninfected erythrocytes and enriched infected cells were washed and identically loaded with Fluo-8 AM (AAT Bioquest, Sunnyvale, CA, USA); the loading buffer contained 150 mM NaCl, 20 mM HEPES, 0.1 mg/mL BSA, 1.0 mM EGTA, pH 7.4 with NaOH. Cells were loaded with 5 μM Fluo-8 AM at a 3% haematocrit for one hour at 37°C in the dark with intermittent resuspension. The cells were then washed and resuspended in the same buffer supplemented with CaCl_2_ or EGTA to the indicated free Ca^++^ concentrations at a final 1.5% haematocrit and 100 μL volume in 96- or 384-well microplates; where present, transport inhibitors were added during this resuspension. Transport inhibitors were obtained from Sigma Aldrich (St. Louis, MO, USA) with the exception of the specific PSAC inhibitor ISPA-28 (a dihydroisoxazole-5-carboxamide derivative from ChemDiv, San Diego, CA, USA). Kinetic fluorescence measurements (excitation 485 nm, emission 528 nm) were then immediately initiated at room temperature. When EGTA or SDS were added during the course of a kinetic experiment, 20x stock solutions were used to minimize effects of dilution; all experimental and control wells were resuspended identically before resuming the kinetic microplate readings.

Fluo-8 efflux from uninfected erythrocytes was measured after loading the dye as above. Cells were resuspended at 1.5% haematocrit in the above saline with 1.5 mM CaCl_2_, 0.5 mM EGTA, and either 0 or 5 mM probenecid (4-(dipropylsulfamoyl)benzoic acid, Sigma Aldrich, St. Louis, MO, USA); this solution has a buffered free Ca^++^ concentration of 1.0 mM. The cell suspension was incubated in the dark at room temperature. At 30 min intervals, a fraction of the cell suspension was harvested and centrifuged to remove the cells. At each time point, triplicate fluorescence measurements on the supernatant were made as above to quantify dye efflux.

### Fluorescence confocal microscopy

Uninfected and enriched Dd2-infected erythrocytes were loaded with 5 μM Fluo-8 AM as above using a modified loading buffer containing 150 mM NaCl, 20 mM HEPES, 0.1 mg/mL BSA, 1.0 mM CaCl_2_, 10 mM probenecid, pH 7.4 with NaOH. After washing, the cells were transferred to cover glass bottom dishes (MatTek, Ashland, MA, USA) and visualized on a Leica SP2 laser scanning confocal microscope (Leica Mircosystems, Exton, PA, USA) under a 68X oil immersion objective. Images were processed using Imaris 7.6.5 (Bitplane AG, Zurich, Switzerland) and uniformly deconvolved using Huygens Essentials 4.5.1 (Scientific Volume Imaging BV, Hilversum, The Netherlands).

### Parasite growth inhibition assays

To evaluate parasite requirement for extracellular Ca^++^, the Dd2, HB3, and 3D7A parasite lines were cultivated in media with varying external free Ca^++^ concentrations. SYBR Green I nucleic acid dye was used to measure parasite growth as described previously [15]. Sorbitol synchronized ring-stage parasite cultures were seeded in 96-well microplates at 0.5-1.0% parasitaemia and 2.0% haematocrit in media supplemented with EGTA at varying concentrations between 0 and 2 mM. These cultures were then maintained at 37°C for 72 hours without medium changes prior to lysis in 20 mM Tris, 10 mM EDTA, 0.016% saponin, 1.6% triton X100, pH 7.5 with 2x Sybr Green I (Invitrogen, Carlsbad, CA, USA). After a 30 min incubation at room temperature in the dark, parasite nucleic acid production was quantified with triplicate fluorescence measurements (excitation, 485 nm; emission, 528 nm). Growth was normalized to 100% for no EGTA addition after subtraction of background fluorescence associated with matched cultures killed with 20 μM chloroquine. In each experiment, EGTA toxicity due to mechanisms other than Ca^++^ chelation was evaluated by equimolar addition of CaCl_2_ during the 72 hour cultivation.

### Measurement of free extracellular Ca^++^ concentrations

Free Ca^++^ concentrations in culture media with and without EGTA addition were measured with an ion sensitive electrode (Cole-Parmer, Vernon Hills, IL, USA). Freshly prepared Ca^++^ standards were used to estimate an electrode slope of 27 mM/decade, which follows Nernstian predictions and indicates electrode specificity for Ca^++^. These measurements were used to determine the free extracellular Ca^++^ concentrations required to support *in vitro* parasite propagation.

### Statistical methods

Pairwise comparisons used the Student’s *t* test to evaluate statistical significance. Assay reproducibility in 384-well microplates was estimated using the *Z’* statistic [29]. This scalar parameter was calculated according to $Z'=1-3\cdot \left[\frac{\left(\sigma_{\mathrm{pos}}+\sigma_{\mathrm{neg}}\right)}{\left(\mu_{\mathrm{pos}}-\sigma_{\mathrm{neg}}\right)}\right]$ where *σ*_*pos*_*, σ*_*neg*_*, μ*_*pos*_ and *μ*_*neg*_ are the standard deviations and mean values of positive and negative control wells.

## Results

### Kinetic measurements of parasite-induced Ca^++^ uptake in microplate format

To allow continuous tracking of the parasite-induced Ca^++^ permeability, it was important to develop a microplate-based assay. Several fluorescent Ca^++^ sensitive dyes were surveyed before selecting Fluo-8, a bright dye that overcame quenching by haemoglobin and yielded suitable detection in erythrocytes [30,31]. The Ca^++^ affinity of Fluo-8, reported at 389 nM, was also attractive because it should produce a low signal from uninfected erythrocytes, which maintain low intracellular free Ca^++^ concentrations through the action of a Ca^++^ ATPase pump [32].

**Figure 1:**
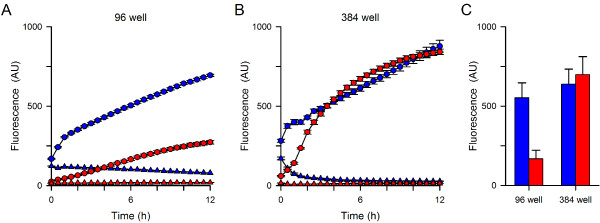
**Kinetics of fluorescence development. (A)** Fluorescence kinetics in 96 well format. Symbols represent means ± S.E.M. of replicate wells for Dd2-infected and uninfected cells (blue and red symbols, respectively) in 1 mM free external Ca^++^ or 1 mM EGTA (circles and triangles, respectively). Notice greater fluorescence with Ca^++^ than EGTA for both cell types. A difference between infected and uninfected cells is apparent. **(B)** Identical experiment using 384 well format. While fluorescence development is specific for Ca^++^, the greater permeability of infected cells is not clearly resolved in this format. **(C)** Mean ± S.E.M. fluorescence after 12 hours with 1 mM free Ca^++^ for Dd2-infected and uninfected cells (blue and red bars, respectively) in indicated formats. *n* =4-8 independent trials each; *P* = 0.01 and 0.7 for comparisons of infected vs. uninfected cells in 96- and 384-well formats, respectively.

Uninfected or enriched trophozoite-stage infected cells were loaded with the acetoxymethyl ester derivative (Fluo-8 AM), washed, and resuspended into 96-well and 384-well plates with 1 mM EGTA or a free external Ca^++^ concentration buffered to 1 mM (Figure 1A-B, triangles and circles, respectively). Fluorescence kinetics were then monitored to track Ca^++^ uptake by uninfected or infected cells (red and blue symbols, respectively). These studies showed increasing fluorescence in each microplate format with both cell types when external Ca^++^ is present, but significantly less fluorescence without free Ca^++^ in the extracellular solution, consistent with Fluo-8 fluorescence due to specific Ca^++^ binding. Infected cells exhibited greater fluorescence than uninfected cells in the 96-well format (*P* = 0.01, Student’s *t* test, *n* = 4), consistent with infection-associated increases in Ca^++^ permeability. However, this difference was not apparent in the 384-well format (Figure 1B-C, *P* = 0.70, *n* = 7-8 each). These differing results with microplate format may reflect effects of erythrocyte autofluorescence, detector sensitivity issues, or changes in well geometry. Regardless of the specific causes, they raise concerns about the specificity of the fluorescence signal for erythrocyte Ca^++^ uptake. This concern may also apply to measurements in 96-well format, where the Ca^++^ signal with uninfected cells was greater than expected based on the low Ca^++^ permeability of erythrocytes [33].

### Dye leakage via organic anion transporters and block with probenecid

**Figure 2:**
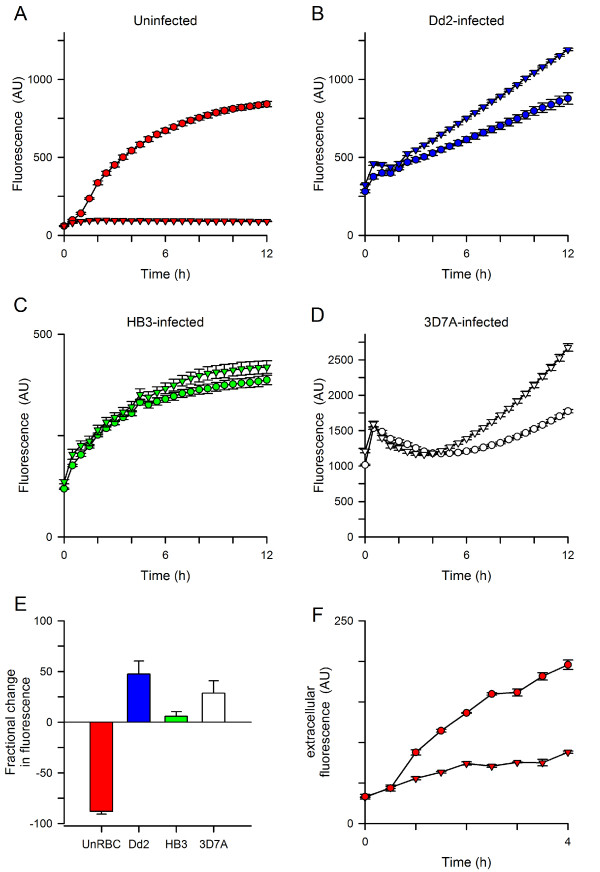
**Effects of probenecid on transport kinetics in 384-well format. (A)** Uninfected cells have a large fluorescence signal in 1 mM Ca^++^ (circles), which is abolished by addition of 5 mM probenecid (triangles). **(B, C, D)** Fluorescence kinetics for erythrocytes infected with indicated parasite lines in the absence or presence of probenecid (circles and triangles, respectively). Note that parasite genotype influences the magnitude of probenecid-associated increase in fluorescence. **(E)** Mean ± S.E.M. effects of probenecid addition at 12 h, normalized to 0% for no effect. *n* = 5-7 trials each; *P* < 10^-5^ and < 0.02 for comparisons of Dd2 to uninfected and HB3, respectively. **(F)** Kinetics of Fluo-8 efflux from uninfected cells in the absence or presence of 5 mM probenecid (circles and triangles, respectively). Error bars, S.E.M. of replicates; representative of two experiments.

Hydrolysis of acetoxymethyl ester groups on the dye by intracellular esterases renders the dye Ca^++^-sensitive; removal of these esters also reduces membrane permeability and is expected to trap the dye within cells. However, organic anion transporters (OATs) on the plasma membranes of many cell types can allow dye leakage; with Fluo-8, this may yield an unwanted fluorescence signal as externalized dye can bind Ca^++^ in the extracellular solution. Probenecid, a broad-spectrum inhibitor of OATs [34], almost completely abolished the signal associated with uninfected erythrocytes (Figure 2A, triangles, 5 mM), suggesting that transporter-mediated Fluo-8 leakage also occurs in this system. Dose-response studies yielded an estimated *K*_*0.5*_ of 0.7 mM for probenecid’s effect on uninfected cells, but no quenching of Fluo-8 fluorescence in control cell-free experiments.

Remarkably, in contrast to the reduced signal seen with uninfected cells, addition of probenecid increased fluorescence in experiments using infected cells (Figure 2B, triangles). This effect of probenecid depended on the specific parasite line used, with Dd2 parasites producing larger increases than the HB3 line; 3D7A produced an intermediate increase (Figures 2B-E, *P* = 0.016 for Dd2 vs. HB3, *n* = 6-7 each). This increased signal may result if probenecid alters the distribution of either Ca^++^ or the Fluo-8 dye within parasite compartments. This explanation is consistent with the known differences in probenecid effects on digestive vacuolar transport in drug-sensitive and resistant parasite lines [35]. It is also consistent with imaging studies that have shown greater trapping of Ca^++^ indicator dyes in the parasite than in host cytosol [24]. Most importantly, a clear difference in Ca^++^-dependent fluorescence kinetics between infected and uninfected cells becomes apparent upon probenecid addition.

To further examine the effect of probenecid on putative organic anion transporters at the erythrocyte membrane, Fluo-8 export from uninfected cells was quantified. Erythrocytes were loaded with Fluo-8 AM, washed, and resuspended in dye-free buffer with 1 mM free Ca^++^. At timed intervals, a fraction of the suspension was centrifuged to remove cells and harvest the supernatant for fluorescence measurements. This experiment revealed increasing Fluo-8 content in the extracellular solution (Figure 2F, circles); 5 mM probenecid inhibited this increase by 70% (Figure 2F, triangles), providing additional evidence for transporter-mediated dye export at the host membrane. The gradual leak of Fluo-8 in the presence of probenecid may reflect either incomplete block of OATs by the reversible inhibitor or mechanical shearing of cells during resuspension and centrifugation.

**Figure 3:**
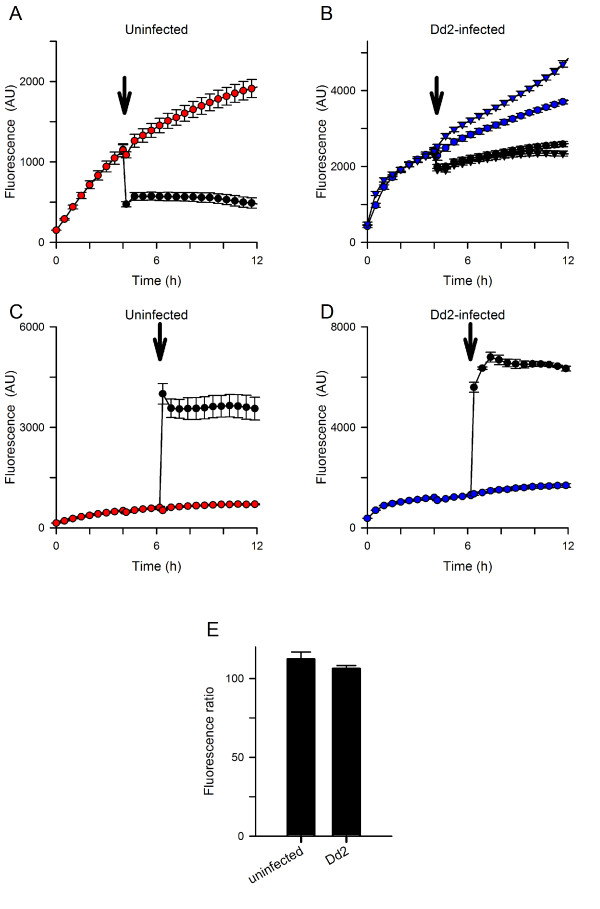
**Effects of EGTA and SDS. (A, B)** Addition of EGTA to chelate extracellular Ca^++^ 4 hours after initiating uptake (arrow in each panel; final [EGTA], 3.33 mM); the free Ca^++^ was buffered at 1.0 mM prior to EGTA addition. Panel **A** shows uninfected cells in the absence of probenecid. Here, EGTA addition abruptly reduces the fluorescence and prevents further increases (black circles); control kinetics are shown for resuspension of cells without addition of chelator (red circles). Panel B shows matched experiments for Dd2-infected cells in both the presence and absence of 5 mM probenecid (triangles and circles, respectively). With infected cells, the initial reduction with EGTA addition is smaller (black symbols), suggesting a lesser contribution of exported Fluo-8. EGTA prevents subsequent increases in fluorescence; probenecid-induced augmentation is also abolished (compare black triangles to black circles). These processes depend on extracellular Ca^++^ and uptake at the host membrane. **(C, D)** Addition of SDS to lyse cells 6 hours after initiating uptake with uninfected and Dd2-infected cells in the absence of probenecid (arrows; final [SDS], 0.05%). Note the large increases in fluorescence after addition of SDS (black circles), when compared to resuspension only kinetics (red or blue circles). **(E)** Effect of probenecid on plateau fluorescence after SDS addition, calculated by normalization of matched controls without probenecid to 100%. Bars represent mean ± S.E.M. of replicates from 2-3 independent experiments.

Extracellular addition of EGTA, a high-affinity Ca^++^ chelator, rapidly reversed the fluorescence signal associated with uninfected cells in the microplate-based assay (arrow and black circles, Figure 3A). The abrupt and large decrease in fluorescence in this experiment is consistent with a signal determined primarily by externalized Fluo-8: the added EGTA will successfully complete with Fluo-8 for binding to extracellular Ca^++^ and therefore reduce fluorescence. After this abrupt decrease, the fluorescence continued to decrease with slow kinetics. The extent and kinetics of signal reversion are complex, depending on the concentrations of EGTA and Fluo-8, their relative affinities for Ca^++^, and unbinding rate constants. In contrast, extracellular addition of EGTA to microplate wells containing infected cells produced a much smaller reduction in fluorescence (Figure 3B); its magnitude was not affected by probenecid. These observations suggest a more modest contribution of externalized Fluo-8 to the fluorescence signal in infected cells. Extracellular EGTA abolished further increases in fluorescence and also inhibited augmentation by probenecid (Figure 3B), suggesting that Ca^++^ uptake at the host membrane is required for both processes.

Addition of SDS to permeabilize membranes and release intracellular Fluo-8 produced large increases in fluorescence with both uninfected and infected cells (black circles, Figures 3C and D), indicating comparable loading and hydrolysis of Fluo-8 AM by these cell types. This observation also suggests relatively low Ca^++^ contents within host and parasite compartments. The steady-state fluorescence after SDS addition was not significantly influenced by probenecid addition (*P* > 0.1 for both uninfected and infected cells, Figure 3E), suggesting that this inhibitor does not directly affect dye fluorescence associated with Ca^++^ binding.

### Confocal fluorescence microscopy confirms the increased Ca^++^ permeability of infected erythrocytes

**Figure 4:**
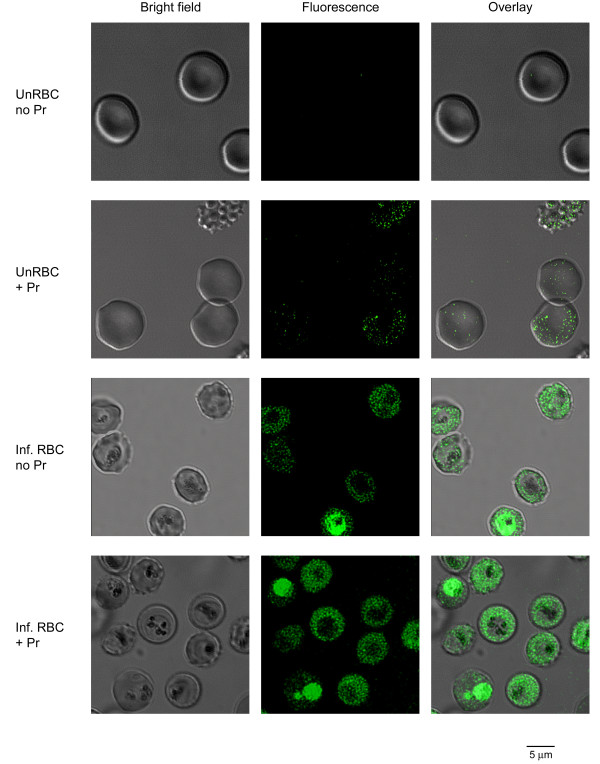
**Localization using confocal microscopy.** Bright field, fluorescence, and overlay DIC images of uninfected and infected cells loaded with Fluo-8 AM (top and bottom pairs of rows, respectively). Cells were loaded in the presence or absence of probenecid (Pr) and imaged in media containing 1 mM Ca^++^. Cell-associated fluorescence is not detected in uninfected cells in the absence of probenecid, consistent with exported Fluo-8; probenecid reveals a weak intracellular signal. With infected cells, a clear signal is detected within both host and parasite compartments. Cell-to-cell variability prevented quantitative assessment of probenecid effect.

Confocal microscopy was then used to examine dye localization and possible mechanisms for probenecid’s effects on fluorescence kinetics. In both the absence and presence of probenecid, there was negligible fluorescence associated with uninfected erythrocytes (Figure 4, top panels). This observation is consistent with a low Ca^++^ permeability of uninfected human erythrocytes and the above studies, which suggest that externalized Fluo-8 accounts for the large signal in the absence of probenecid. On the other hand, imaging of infected cells revealed fluorescence in both the host and parasite compartments, regardless of whether probenecid was included (Figure 4, bottom panels). A relatively homogeneous fluorescence signal was observed within the host erythrocyte compartment of infected cells. There was however a variable signal associated with the intracellular parasite. This variability may reflect effects of parasite stage-dependent dye loading or Ca^++^ transients associated with specific developmental events; large Ca^++^ transients are associated with cell cycle regulation in other eukaryotes [36]. This variability hindered attempts to examine how probenecid increases fluorescence signals in infected cells. Nevertheless, these findings confirm the greater Ca^++^ permeability of infected cells; they also implicate rate-limiting Ca^++^ uptake at the host erythrocyte membrane in the kinetic microplate assay (Figures 2 and 3).

### The parasite-induced Ca^++^ permeability is distinct from PSAC and mammalian Ca^++^ channels

**Figure 5:**
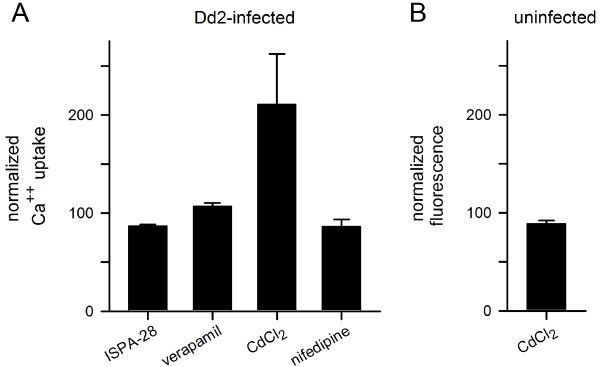
**Effects of known transport inhibitors. (A)** Mean ± S.E.M. Ca^++^ uptake by Dd2-infected cells in the presence of 15 μM ISPA-28, 10 μM verapamil, 100 μM CdCl_2_, or 10 μM nifedipine, normalized to 100% for control measurements over 12 hours without inhibitor. Cd^++^ increases parasite-associated fluorescence, but none of the agents block the parasite-induced erythrocyte Ca^++^ permeability. **(B)** Mean ± S.E.M. fluorescence change associated with 100 μM CdCl_2_ for uninfected cells in media without probenecid. At these concentrations, extracellular Cd^++^ does not contribute significantly to the signal.

The above findings suggest that the parasite induced Ca^++^ permeability of infected cells can be studied in isolation if host OATs are inhibited with probenecid. With these precautions, Fluo-8 was then used to examine possible inhibitors of the Ca^++^ uptake mechanism. ISPA-28, a potent and specific inhibitor of PSAC activity in Dd2-infected cells (*K*_*0.5*_ = 56 nM) [14], had only a modest effect on the Ca^++^-dependent fluorescence despite use of a high 15 μM concentration (Figure 5A), excluding significant uptake of Ca^++^ via PSAC. The modest inhibition observed may reflect block of slow Fluo-8 efflux through PSAC; more direct studies of organic dye transport via PSAC may be warranted.

Known inhibitors of mammalian Ca^++^ channels were also examined; 10 μM verapamil, 100 μM CdCl_2_, and 10 μM nifedipine all failed to inhibit infected cell Ca^++^ uptake (Figure 5A). Notably, CdCl_2_ significantly increased fluorescence in infected cells, possibly by altering distribution of either Ca^++^ or Fluo-8 in parasite compartments. CdCl_2_ did not alter the signal associated with uninfected cells (Figure 5B), excluding a fluorescence artifact resulting from Cd^++^ binding to externalized Fluo-8 at the divalent cation concentrations used here. Importantly, the effect of Cd^++^ on infected cell signals serves as a positive control for future small molecule inhibitors and agonists of the parasite-induced Ca^++^ transport pathways at the host membrane. These findings suggest that the parasite-induced pathway has pharmacological properties distinct from both PSAC and mammalian Ca^++^ channels [37].

### Ca^++^ uptake is required for intracellular parasite development

**Figure 6:**
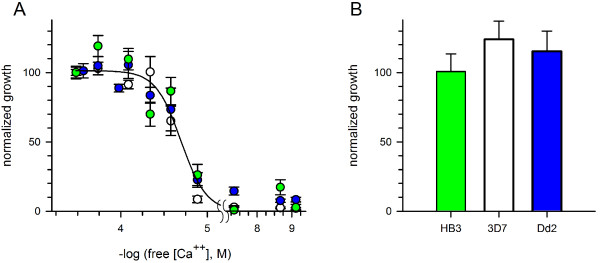
**Conserved parasite requirement for extracellular Ca**^**++**^**. (A)** Normalized growth of divergent parasite lines over 72 hours as a function of measured free external [Ca^++^], achieved by EGTA addition to standard media. Absissca is shown on log scale. Symbols represent mean ± S.E.M. growth of HB3, 3D7A, and Dd2 lines (green, white, and blue circles, respectively). Solid line represents the best fit to a sigmoidal decay. **(B)** Mean ± S.E.M normalized growth of each line when 2 mM CaCl_2_ is added to the medium with 2 mM EGTA, the highest used in panel **(A)**.

To determine if the increased Ca^++^ uptake is required for growth of the intracellular parasite, three *P. falciparum* lines were next cultivated in media with free Ca^++^ buffered to a range of concentrations through the addition of EGTA. Parasite growth over 72 hours was quantified and is presented against the free Ca^++^ in each medium (Figure 6A). These studies revealed that all three parasite lines require extracellular Ca^++^ with indistinguishable dose-response profiles. The relatively low *EC*_*50*_ for the development of these lines (21 ± 1.0 μM) suggests one or more high-affinity transport mechanisms used by the parasite to scavenge Ca^++^. Equimolar addition of Ca^++^ during cultivation with the highest concentration of EGTA fully restored growth of each parasite line (Figure 6B), indicating that EGTA is not directly toxic.

## Discussion

**Figure 7:**
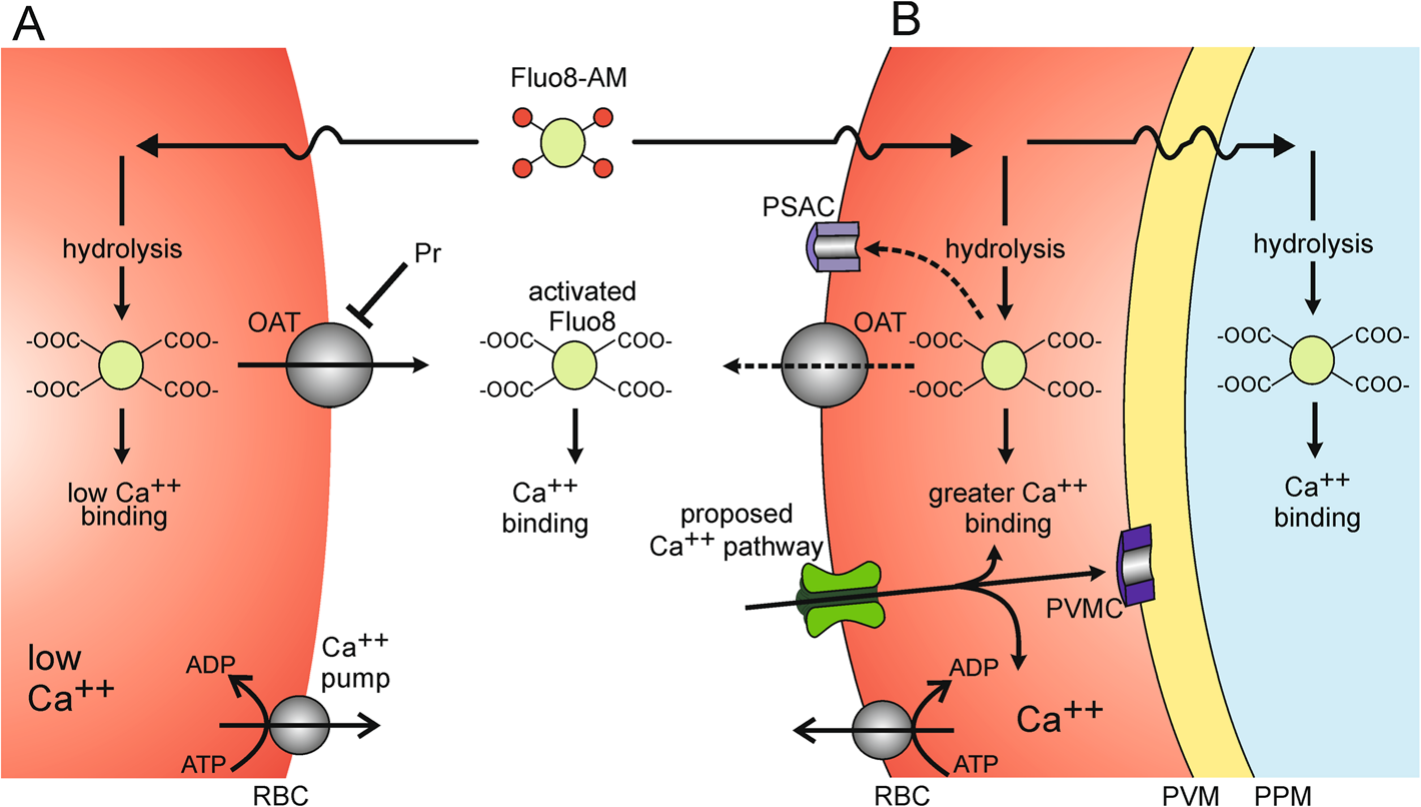
**Schematic showing proposed Ca**^**++**^**and Fluo-8 transport mechanisms. (A)** Uninfected human erythrocytes maintain a low intracellular Ca^++^ through a low passive Ca^++^ permeability and an ATP-dependent extrusion pump (Ca^++^ pump). Fluo-8 AM enters cells by diffusion and is hydrolyzed to yield activated Fluo-8, which fluoresces upon Ca^++^ binding. Fluo-8 may be exported via organic anion transporters (OAT). **(B)** Infected cells have a higher intracellular Ca^++^ through activation of a distinct Ca^++^ uptake pathway. Ca^++^ that enters the cell may be exported via the Ca^++^ pump, may remain in the host cytosol, or may enter parasite compartments by crossing the parasitophorous vacuolar membrane (PVM) through nonselective PVM channels (PVMC) [39,40] and the parasite plasma membrane (PPM) via unknown mechanisms. Fluo-8 AM that enters infected cells may be hydrolyzed to the active form in various compartments; the activated dye may be exported via the host cell OAT and possibly via PSAC. Transport of the dye across parasite membranes may also occur, but is not illustrated.

While documented in multiple studies, the mechanism of increased Ca^++^ uptake by erythrocytes infected with malaria parasites remains poorly understood. Here, Fluo-8, a relatively new and brighter Ca^++^-sensitive dye, has been used for kinetic uptake measurements in microplate format. The findings, when combined with confocal fluorescence imaging, suggest complex facilitated transport of both Fluo-8 and Ca^++^ across infected erythrocyte membranes (Figure 7). Fluo-8 can be loaded into infected erythrocytes via an esterified derivative, Fluo-8 AM, which crosses membranes freely and undergoes hydrolysis by intracellular esterases. This cleavage yields a hydrophilic dye that is normally trapped within the cellular compartment; as with some other cells [38], human erythrocytes can export this dye via probenecid-sensitive organic anion transporters (OAT and Pr, Figure 7). Interestingly, while probenecid reduced fluorescence in experiments with uninfected cells, this inhibitor yielded increased Fluo-8 fluorescence in infected cells; the magnitude of this increase varied significantly with parasite genotype. Because probenecid did not alter Fluo-8 fluorescence in control cell-free experiments, it is likely that this non-specific inhibitor alters dye and/or Ca^++^ transport within parasite compartments. Probenecid may also have additional effects on unrelated parasite activities.

Uninfected erythrocytes maintain a low intracellular Ca^++^ by keeping Ca^++^ permeability very low [33]; Ca^++^ that leaks into the cell is also efficiently extruded by an ATP-dependent pump (Ca^++^ pump, Figure 7) [41,42]. The present studies suggest that infected erythrocytes have higher passive permeability to Ca^++^ via a new pathway on the host cell membrane. Because ISPA-28 does not prevent Ca^++^ uptake, this pathway appears to be distinct from the broad selectivity PSAC. Although the increase in permeability is sufficient to overcome active extrusion by the erythrocyte Ca^++^ ATPase pump [43], its absolute magnitude is small when compared that of excitable cells [24]. This relatively low rate has aggravated electrophysiological studies that might otherwise yield mechanistic information [24,26]. The miniaturized assay reported here overcomes this limitation and yields a robust signal specific for the parasite-induced Ca^++^ permeability. The slow Ca^++^ uptake kinetics, as evidenced by fluorescence development over hours, are consistent with a modest increase in Ca^++^ permeability after infection. This permeability appears to be essential as removal of external free Ca^++^ inhibits growth of the intracellular parasite [21,44]; the present studies have quantified this Ca^++^ requirement and determined that it is conserved in *P. falciparum* parasite lines.

Although multiple transporters on various membranes of the infected erythrocyte may contribute to the shape and kinetics of the fluorescence signals recorded with Fluo-8, Ca^++^ transport at the host membrane is the first step and appears to be rate-limiting [10]. For this reason, the fluorescence assay described here may prove to be suitable for high-throughput screening of inhibitors of the parasite-induced Ca^++^ permeability. Similar approaches have been successfully used to find inhibitors of mammalian Ca^++^ channels [45,46]; high-throughput screens have also been used to find potent PSAC inhibitors [47]. Because these screens usually test each compound in only a single microplate well, a critical issue is the quality of the assay and its signal-to-noise ratio, often quantified with a parameter known as the Z’ statistic [29]. The miniaturized Fluo-8 assay exhibited reproducible Z’ values of 0.65 to 0.8 within the first 2 hours of Ca^++^ uptake, surpassing the generally accepted threshold value of 0.5 for single point screens with low false-positive rates. Inhibitors found through screening should be useful tools for examining the precise mechanisms of transport at the infected cell membrane. They may also be starting points for development of anti-malarial drugs that work by preventing parasite access to Ca^++^ from the host plasma. Drug discovery against this activity is supported by the conserved requirement for extracellular Ca^++^ in parasite lines, the numerous roles that Ca^++^ is thought to serve in parasite biology, observed differences between this pathway and mammalian Ca^++^ channels, and recent genome-wide association studies that have implicated erythrocyte Ca^++^ extrusion as a major determinant of susceptibility to severe malaria [48].

## Competing interests

The authors declare that they have no competing interests.

## Authors’ contributions

EMZ, MN, AK, KR and SAD conceived the project, designed and performed experiments, carried out data analysis, and wrote the paper. All authors read and approved the final manuscript.
